# Online toxic speech as positioning acts: Hate as discursive mechanisms for othering and belonging

**DOI:** 10.1177/14614448251338493

**Published:** 2025-05-27

**Authors:** Esteban Morales, Jaigris Hodson, Victoria O’Meara, Anatoliy Gruzd, Philip Mai

**Affiliations:** University of Groningen, The Netherlands; Royal Roads University, Canada; University of Leicester, UK; Toronto Metropolitan University, Canada; Toronto Metropolitan University, Canada

**Keywords:** belonging, Colombia, online harms, othering, positioning, Telegram, toxic speech

## Abstract

While digital platforms foster a sense of community and identity, they also facilitate harmful exclusionary practices. In this context, toxic and hateful speech are key mechanisms not only for harming others but also marking processes of othering and belonging. In this article, we examine the role of hateful and toxic speech in structuring processes of in- and out-group formation and maintenance by focusing on a public Colombian Telegram group. More specifically, we examine how members use toxic speech to position themselves and others in relation to narratives emerging from the group by analyzing 3221 posts with high levels of toxicity. Our analysis yields insights into the complex and paradoxical uses of antisocial behavior on social media platforms. Overall, the findings of this study deepen our understanding of the social gratifications that underlie how hate and toxic speech are used to disenfranchise individuals.

## Introduction

At the core of people’s uses of popular social media platforms, there is a desire to connect with other like-minded individuals in ways that develop or reinforce users’ identity and belonging (boyd, 2014; Roberts and David, 2020; Smith et al., 2021). However, the role of platforms in establishing healthy bonds in and across communities is not straightforward. While research suggests a positive connection between social bonding and digital platforms, it also reveals destructive dynamics like the rapid spread of misinformation, manipulation campaigns, ideological segregation, and extremism (González-Bailón and Lelkes, 2023). Despite these tensions in platforms’ social role, they have become places through which people express belonging while also setting clear boundaries for those who do not belong (Wilson et al., 2020). In brief, social media platforms are spaces not only to say *I belong to these communities* but also to say *you do not belong here*.

One way participants enact the social processes of belonging and othering is through hateful and toxic speech. On one hand, toxic speech acts as a way to set moral boundaries among members of the in-group. For example, collective harassment often enforces group adherence to moral/normative behaviors, for example, where “a member of a social network accuses a target of violating the network’s norms, triggering moral outrage. Network members send harassing messages to the target, reinforcing their adherence to the norm and signaling network membership” (Marwick, 2021: 4). At the same time, toxic speech can also be a way of identifying and attacking out-group people. For example, social media plays an increasingly central role in supporting and expanding animosity between opposing groups by being a space where in-group members can dehumanize out-group members to justify harming them (Harel et al., 2020). Thus, online toxic speech is one way people connect in the contemporary digital landscape (de Keulenaar, 2023; Rathje et al., 2021). Platforms often let these manifestations of antisocial sociability flourish, as they are a common form of affective polarization that drives user engagement (Nordbrandt, 2023).

As the reasons for toxic speech can often be ambiguous, they require a more nuanced understanding of their enactment across variegated contexts. With this goal in mind, the objective of the current study is to examine the role that toxic speech plays in structuring processes of in-and out-group formation and maintenance. Focusing on a public Colombian Telegram group (“Chismes Frescos Medellin,” or “Fresh Gossip Medellin”) where over 120 K users discuss local news, politics, and sports, we analyze 3221 posts with high levels of toxicity to examine how members use online violence to position themselves and others in relation to narratives emerging from the group (i.e. for or against). Our analysis yields insights into the complex and paradoxical uses of antisocial behavior on social media platforms and deepens our understanding of how toxic speech is used to (dis)enfranchise individuals.

## Literature review: toxic speech as acts of (anti)sociability

While antisocial behavior has been endemic to the Internet (Phillips, 2019), encompassing a wide range of practices, contexts, and technological affordances (Morales, 2023), *toxic speech* has emerged as a particularly pernicious manifestation. Drawing from the metaphor of the harm that toxins cause to our bodies, toxic speech emphasizes how discourse “damages the social body” (Tirrell, 2017: 142) by shaping how we relate in our communities. Toxic speech can take the form of threats of physical harm, insults, and patronizing discourses (Pradel et al., 2024), and it is marked by incivility, intolerance, and intentions of harm (Suarez Estrada et al., 2022). And while toxic speech has long been a part of our everyday discourses, it is a particularly prevalent phenomenon in digital platforms. Here, “the structure of platforms, driven by their economic logic, is the fundamental context in which toxicity emerges, as it allows discursive violence to be quickly spread and legitimated” (Recuero, 2024: 3; see also Munn, 2020).

However, exclusively defining toxic speech by the harm it causes to the social body is reductive and fails to consider the role of social, cultural, and communicative practices in antisocial behaviors (Barth et al., 2023; McCosker, 2014). As Tirrell (2017) explains, “even toxins aimed at medicinal healing inflict damage; when chemotherapy introduces toxins to kill cancer cells, the toxin kills, then the body restores” (p. 142). In practice, this demands an understanding of toxic speech sensitive to specific contexts and the gratifications users derive from enacting and amplifying it.

The social, cultural, and communicative aspects of toxic speech require a more nuanced examination of the spectrum of civility/sociality on social media platforms and how antisocial and prosocial behaviors are developed, enacted, and received by others. On one hand, the harm caused by platforms fomenting and amplifying incendiary and extremist content cannot be diminished (Munn, 2020), as they can impede conflict resolution and de-escalation by promoting processes of othering that contribute to dehumanization and harm (Harel et al., 2020). Indeed, toxic speech is a key mechanism through which social media users demarcate in- and out-group users (Mamakos and Finkel, 2023), silencing and delegitimizing “others” (Suarez Estrada et al., 2022), and positioning them as a clear threat to their (and their group’s) well being (Harmer and Lumsden, 2019).

On the other hand, social media discourses (even harmful ones) have a role in building and sustaining cultural narratives, even turning into spaces to coordinate activism and belonging (Haunschild et al., 2022) among what Nancy Fraser (1990) calls “subaltern counterpublics.” Certainly, it is tolerated—and sometimes even expected—that people will engage in antisocial behavior as a form of retributive justice to control those committing offenses against specific groups (Blackwell et al., 2018). For example, toxic speech has been used by Ukrainians to delegitimize those from Russia who attack their territories. Here, Tovares (2018) shows how online newspaper comments in Russia ridiculing Putin’s height acted as a form of creative insurgency leading to popular resistance by challenging “the existing hierarchies and [delegitimizing] the official (centripetal) forces by ridiculing them” (p. 242). Similarly, McCosker (2014) remarks that trolling (a practice closely related to toxic speech) can be seen as acts of provocation that, at times, enable civic conversations on issues of power. These studies showcase the importance of better understanding the social gratifications of engaging in antisocial behavior and its implications for civil dialogue and well-being.

It is thus exceedingly difficult, if not impossible, to completely separate the social and antisocial dimensions of toxic speech – as their limits are not only incredibly thin but also often coexist simultaneously. As such, determining what is *toxic* and what is *healthy* is mostly determined by the normative values within a particular community, as well as the identities at play and the nature of the toxic utterances (Blackwell et al., 2018; Kim et al., 2022; Schmid et al., 2022). To navigate the paradoxical nature of antisocial behavior and facilitate contextually sensitive framings, Cover (2022) suggests we center examinations of such behaviors through a lens of everyday cultural experiences. That is, by focusing on how antisocial behaviors are enacted within specific cultural and normative contexts, researchers can better understand the strategic use of toxic speech as a double-edged sword that is used to protect in-group members and harm outsiders (Kaakinen et al., 2018; Keller and Askanius, 2020; Marwick, 2021). To be clear, we do not mean to suggest that toxic speech can be excused as prosocial or categorically positive in one context and antisocial in another. Rather, we sustain that it is necessary to create better (political, social, technical) approaches to mitigate toxic speech by illuminating its role in the lives of those who engage in it. After all, if people are getting social, emotional, or psychological gratifications from their toxic speech acts in the community, they will be less likely to respond to interventions intended to limit the toxicity unless we can find another way to support the community building the toxicity affords.

## Theoretical lens: positioning theory

In exploring toxic speech as a social, cultural, and communicative process, we focus on the means through which users navigate and mark their positionality through toxic speech, both as individuals and as part of their preferred community. Drawing on positioning theory, we explore how people use discourse to “locate themselves and others” (Moghaddam and Harré, 2010: 2) within specific social contexts. In doing so, we account for the reality that people often take two different positions simultaneously when speaking in social settings: an exploration of who they are and an examination of who others are (or who they ought to be according to their values). Applied to digital platforms, posting becomes an ongoing process of positioning oneself with and in opposition to others, in addition to the prevailing narratives of the community (Vizcaíno-Verdú and Abidin, 2022). Viewed in this way, toxic speech is an integral part of the way users’ construct identities (Lee et al., 2008; Moghaddam et al., 2008).

Barring a few exceptions (Badarneh and Migdadi, 2018; Labben, 2022; Sadaf and Siitonen, 2022), limited research has explored online antisocial behavior through the lens of positioning and importantly none has focused explicitly on toxic speech. While limited, these studies have emphasized the critical importance of paying closer attention to the discursive constructions of the self and others in understanding the individual, cultural, and social factors that enable, sustain, and expand toxic speech. For example, Sadaf and Siitonen (2022) draw on positioning theory to show how, amid the cultural context of social movements in Pakistan, women who actively engage in protest are deemed ‘other’—and thus marginalized and excluded. Accordingly, in the following sections, we delve into the potential of this theoretical work to shed light on the various ways toxic speech acts within and as a form of cultural and communicative processes.

## Methods

To explore the role of toxic speech in users’ positioning of themselves and others within online communities, we focus on *Chismes Frescos Medellin* (*Fresh Gossip Medellin*), a public Colombian Telegram group for residents of Medellin to discuss everyday local news. This group was chosen for its popularity (125,676 users in October 2023) and the window it provides into Medellin’s unique historical, social, and cultural context. Indeed, as Colombia has long suffered from a trail of armed conflict and waves of violence (Centro Nacional de Memoria Histórica, 2016), Medellin became a sort of microcosm of the dynamics of war in the country – weakening of the social fabric of its communities and destroying the lives of many of its inhabitants (Dávila, 2016; Giraldo-Ramírez, 2008). In this context, violence became a key mechanism for citizens of Medellin to make sense of their surroundings (Martín- Barbero, 2006), including violence on digital platforms (Morales, 2024).

Another important piece of the context of this study is its technological boundaries. Telegram, one of the platforms with the most consistent growth in the last few years (Telegram, 2022), has increasingly become an online space where people gather to share news and socialize. However, several studies have shown that Telegram is also a platform that often hosts violent content and communities (e.g. Walther and McCoy, 2024). As mainstream platforms such as Facebook and YouTube de-platformed users prior to 2024 for engaging in antisocial behavior and other violations of their Terms of Service, many of those users migrated to Telegram (Rogers, 2020). In this context, Telegram is often seen as strongly opposed to censorship and content moderation, a place for contentious politics and statements without fear of silencing or reprimanding (Wijermars and Lokot, 2022).

### Data collection and preparation

Data were collected using Communalytic, a computational social science research tool for studying online communities and discourse (Gruzd and Mai, 2023). We collected 98,729 publicly available posts from the Telegram group shared between April 2023 and September 2023. Next, we used Communalytic’s built-in Civility Analyzer module, which employs Detoxify – a machine learning–based Python library – to identify posts likely containing toxic speech. The module examines linguistic patterns and contextual features to assign probability scores ranging from 0 (least toxic) to 1 (most toxic) across categories such as insults, threats, and identity-based attacks, based on predefined human annotations (Unitary Team, 2020). For this study, we focused on the broadest category, “toxicity,” to capture a wide range of toxic expressions, from subtle insults to overt hate speech, which may be perceived as rude or disrespectful by Telegram users. Following prior research (Gruzd et al., 2020; Pascual-Ferrá et al., 2021), only posts that received a toxicity score of 0.7 or above were included in the final dataset (n = 3221), as posts within this range are likely to be perceived as toxic by a majority of users.

Importantly, like Pohjonen and Udupa (2017) and Barth and colleagues (2023), we recognize that identifying toxic and potentially harmful language is not a mere technical process. Instead, it can only be fully understood when framed within the cultural and normative values of the community. Because of this, after the automated analysis, we conducted a manual review of the toxic posts flagged by Detoxify. This manual analysis provided a deeper understanding of the cultural and normative conditions of the community we analyzed. Upon manual review of the 3221 posts, 66 posts were deemed to be misclassified and subsequently removed as they did not contain hateful or toxic speech, resulting in a final sample size of 3155 posts (~3.2%). This sample was further analyzed manually using thematic analysis to capture the context in which toxic expressions were used by this community.

Finally, we relied on the constant comparison method (Strauss and Corbin, 1990) to identify and focus our qualitative analysis on a subset of the toxic messages that represent the most salient topics. In total, we identified four prevalent topics accounting for nearly 40% of our dataset: (1) security (n = 652, 20.2%); (2) politics (n = 252, 7.8%); (3) migration (n = 188, 5.8%); and, (4) gender (n = 166, 5.2%). Approximately 30% (n = 934) of the remaining posts in our dataset were dedicated to personal attacks on group members without a clear narrative, with the rest falling into less discussed narratives, such as animal rights (n = 69, 2.1%) and animosity between football fans in the city (n = 28, 0.9%). We focus our subsequent analysis on the 1258 toxic posts concerning the four main topics (security, politics, migration, and gender) found in our sample.

### Trigger warning

While this article examines toxic and potentially harmful speech as a communicative and cultural process, over-emphasizing the sociability of online abuse can obscure its ethical and moral dimensions. In response, we argue that acknowledging the social and cultural dimensions of antisocial behavior is not inherently an effort to minimize its harm, but calls for its deliberate and careful analysis. To mitigate this risk, we adopt Jane’s (2015) approach by including direct instances of toxic language rather than relying on vague generalizations. In the following sections, we present direct quotations from participants’ conversations that include crude and offensive language, as well as threats of physical violence and abuse. We want to extend a trigger warning for readers – and especially those who have experienced abuse or violence – as such content may evoke past trauma.

### Data analysis

To respond to our research question, we relied on thematic analysis (Braun and Clarke, 2022) to code the data according to two key aspects of our theoretical lens (i.e. positioning theory). First, we categorized who was being positioned through the toxic speech using three themes: (1) *self*, where we considered efforts by users to position themselves (e.g. “I crush all those Venezuelan sons of bitches”); (2) *other*, where we considered efforts by users to define and position the *other* in relation to a given narrative (e.g. “These women of today do not even make me angry haha they are getting ridiculous with such feminism”); and, (3) *the group*, where we considered users’ efforts to position the normative boundaries of the group, often by replying to each other (e.g. “What a stupid comment, he must not have a mom or sisters, how disgusting a guy like this”). When assessing toxic messages in relation to the three themes (self, other, and group), we paid particular attention to so-called *positioning acts;* that is, identifying how people negotiate their positionalities across the conversation (Day and Kjaerbeck, 2013). As recommended by Braun and Clarke (2022), we do not present positioning acts as independent “boxes” that stand apart from one another. Rather than merely counting how often specific themes appeared, we focused on capturing the nuanced meanings and contextual subtleties within the data. This allowed us to narrativize these themes to better showcase how they flow across the sample while capturing the qualitative nuances of group members’ positioning acts.

The coding was done by the lead author through an iterative process in close consultation with the research team, continuously refining the codes to maintain the interpretive depth of our findings. The findings are organized based on the four main topics (security, politics, migration, and gender) identified in our sample.

## Findings

### (In)security

In this theme, group members often relied on toxic speech to position themselves as victims or possible vigilantes concerning insecurity in the city. Posts often recalled instances where community members were targets of criminality (e.g. “This rat was going to rob me”), frequently accompanied by calls for action (e.g. “We have to catch the rats and beat them”). As these two exemplars showcase, accompanying the positioning of the other are often statements that serve to dehumanize those who are seen as criminals, calling them pests or rats. Emojis also frequently contain images of rats or bugs to refer to people outside the group. Consequently, many in the group relied on toxic speech to position themselves as being against a criminal other, often violently so. For example, when discussing a robbery, a participant responded, “I would throw my car at them.”

Along with these acts of identifying the self concerning crime, there was an effort to position others in the city as responsible for the insecurity. In some cases, participants actively aimed to position others – often not group members – as criminals (e.g. “This is one of the rats that steals cell phones during rush hour in the subway”). Accompanying this effort to position others as responsible for insecurity comes an assumption of what they deserve (violence). For instance, a participant noted: “Let every thief they catch be killed, and they will be finished.” Another one argued: “Let us fumigate those damned scum at once.” Through these discursive mechanisms, participants outline a clear *other* who is sub-human and deserves to be severely punished for the societal harms they might cause.

Alongside the discursive mechanisms positioning the other (as criminals) and the self (as either victims or vigilantes), participants often relied on toxic speech to position the Telegram group within specific normative and moral boundaries. For instance, when toxic speech was used to impose the narrative that criminals in the city deserve to be killed on the street (e.g. “That scumbag deserved to die”), any poster who disagreed was often heavily criticized. For example, when somebody questioned the fairness of skipping due process to prosecute criminals, a participant replied, “It is not fair? That is little for what a scum like that deserves, and the law here in Colombia does not exist.” As this narrative advanced, there were increasing calls to highlight whose narratives belonged in the group (e.g. “I totally agree, a depraved person like that deserves death before he does something worse”) and who did not belong in the group, often through direct threats and insults (“You are a danger to society” and “I hope you die fast, dog”). Through such toxic language, participants marked members of the Telegram group who did not align with the dominant group perspectives as contributors to and even responsible for the city’s insecurity and criminality.

### Political (dis)affiliation

Unlike the (in)security narrative, we find that efforts to position *self* and *other* in the context of political issues are fickle. That is, while toxic posts related to (in)security were primarily intended to categorically denounce those who engage in criminal acts, they rarely explicitly defend their position (i.e. anti-criminal) as “best” or “correct” relative to other perspectives. For example, two participants from opposite ends of the political spectrum may agree that (in)security is a pressing problem but use toxic speech to position the *other*’s political ideology as ignorant and complicit in perpetuating the problem. Such positioning was evidenced across the political spectrum: right-leaning users insulted those who supported leftist politics, including the current president (“Bastard president and those who voted for that rat”); left-leaning users identified those with right-wing views as thieves (“another one of the thousands who don’t know that Duque [a right-wing ex-president] was the one who stole 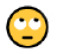
”); and users who self-identified as apolitical targeted everybody defending politics (“both leftists and rightists no longer have the right to criticize, all politicians are crap”).

As such, the political (dis)affiliation narrative highlights how the positioning of both the *self* and *other* in the Telegram group are mutually informative and subject to constant (de)construction at the group level. Indeed, participants continuously employed toxic speech to establish moral boundaries by rejecting the opinions of those who thought differently. For instance, one participant wrote of another: “It is known that she is a leftist brute. Don’t ask for more from that dumbass.” Another participant argued, “don’t come here [the Telegram group] to say that in 10 months Colombia has already been damaged. When they [right-wing political party of the ex-president] handed over the government, everything was already turned to shit 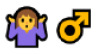.” Through these toxic statements, participants show how political affiliation is often positioned as a moral boundary that limits belonging and othering – a dividing line that is continuously drawn and renegotiated through the use of toxic speech.

### Migration

In this theme, we find that users often seek to position migrants as responsible for the problems facing Medellin (e.g. “Most Venezuelans came to this country to do damage. They should go back to their filthy country 
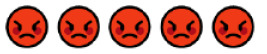
.”) and dehumanize them (e.g. “They [migrants] are parasites and that is why they damaged the whole system of their country and did not fight for it, cowardly Venezuelan rats.”). Much like the (in)security narrative, toxic speech concerning migrants includes statements about what migrants deserve (e.g. “What I want most is for all Venezuelan thugs, thieves and all the bad guys to emigrate”), advocating for physical harm (e.g. “Venezuelan rats. Too bad the driver didn’t have a gun and give those assholes some lead”), and positioning themselves as victims of egregious behavior by migrants (e.g. “I hired [a Venezuelan] and it was the worst thing I did, he left with the competition after I picked him up off the street because he was eating a cable. Now he is my number 1 enemy. The day I catch him I will give him misery. They are rats.”).

While some users in our sample used toxic speech to challenge narratives that *other* and dehumanize migrants (e.g. “I had Venezuelans working for me . . . and I have many, many Venezuelan friends and they are all excellent people. Stop the stupidity and don’t generalize.”), we found that pro-migrant statements that employed toxic speech were met with anti-immigrant statements employing the same toxic speech (e.g. “Obviously there is no shortage of those who have a Venezuelan rat partner and defend them, but whatever they say they will always be a pest, a plague, undesirable parasites. 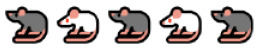.”). Overall, group members engaged in these speech acts intending to position the Telegram group within a normative boundary that recognizes migrants as guilty, less-than-human *others*, rejecting alternative narratives and positionings that favor migrants.

### Gender and sexuality

Finally, discussions about gender and sexuality reveal tensions in how individuals position themselves and others. Some use toxic speech to portray themselves as opposed to gender equality, while others use it to depict themselves as champions of gender equality, even willing to intervene as bystanders to ensure women and members of the LGBTQ+ community are safe. Indeed, on the one side, there were efforts to use toxic speech to position women, particularly feminist women, as *other* (e.g. “They are being ridiculous with their feminism hahaha. You can’t even see them or say hello to them. Glass generation 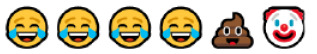
”). This type of othering was also directed at members of the LGBTQ+ community. For instance, when a video was shared with a description that said that a security guard was beating two gay men, a participant responded, “Well done by the warden. What a mess that pair of sissies hahaha.” Along with these efforts of othering women and LGBTQ+ people, group members relied on toxic messages to objectify feminized and queer bodies and minimize the value of their lives. For instance, when a user shared a photo of an alleged robbery conducted by a woman, another group member replied: “What an ass on that thief.” Concerning another post, a member argued: “But it’s a trans fight. That’s worth shit, let the queers kill each other.”

Some group members used posts to call out those committing gender and sexual violence. For example, when somebody shared a video of a man hitting a woman, a user responded, “How nice it would be to knock those teeth out with three kicks to that head. Since he is so macho 
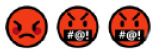
.” In this context, the self is often positioned as aligning with or in defense of these identities through toxic speech, showcasing efforts to tell experiences against those targeting women and members of the LGBTQ+ community, as a group member who replied to somebody calling women “gold diggers,” arguing the following: “Don’t be ignorant, you are being an embarrassment 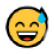.” In line with this, some users position themselves as both capable and looking forward to enacting violence against those who commit abuse against women. For example, a user argued, “I’m eager to see an abuse to chop them [the abusers] one by one 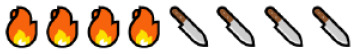 bad life for rapists.” This example showcases how people’s expressions of toxic speech can then signal intentions of bystander intervention to respond to gendered violence in the streets of Medellin – serving as a form of violent counterspeech.

Through these discursive movements in defining the self and the other and what they deserve, group members engaged in toxic speech to negotiate the group’s position within normative boundaries. Importantly, we noticed several instances of users engaging in toxic speech to challenge those engaging in gender and sexual violence, especially concerning the objectification of women. Here, a participant argued, “They can’t see a woman’s profile because they pretend to be don juanes and worse. They are happy sending pictures of their miseries; do not humiliate yourselves, gentlemen.” Through these positioning acts, conversations about gender and sexual violence showcase how toxic speech is used to negotiate and challenge the construction of the other and the normative boundaries of the group, continuously pushing what they perceive as the correct values of the group.

## Discussion

By identifying which interlocutors are accepted, considered other, or seen as outsiders in the Telegram group, our study showcases how toxic speech is used to define and reinforce individual and group positionality. We find that the paradoxical use of toxic speech – used both to reinforce boundaries of what the Telegram group expects and will accept and to push back against these normative boundaries – functions as a constant (re)negotiation of the boundaries of group membership and positionality. Interestingly, we observed two different trends in how these group boundaries were (re)negotiated. For the themes of *(in)security* and *migration*, we witnessed a strong policing of group boundaries and a relatively cohesive sense of what the group will accept. In contrast, we witnessed a greater in-group tolerance for dissent in the *political (dis)affiliation* and *gender and sexuality* themes. We review these trends more closely below.

### (In)security and migration

When using toxic speech acts to establish a sense of security, participants in *Chismes Frescos Medellin* engage in denying the humanity of those seen as criminals, flattening the world into a binary of good people (victims/vigilantes) and evil people (criminals) who deserve the harshest punishment. There is no room within the group boundaries to negotiate other positions – as this Telegram community firmly polices any other approach to understanding criminality, putting it outside the boundaries of acceptable speech. This act of positioning the other as the one to blame for all community problems is a common discursive practice used in various global contexts to profile migrants (see, for example, de Noronha, 2015; Gruzd et al., 2024; Tagwirei, 2016; Webster, 1997). Identifying a criminal “other” does important work related to positioning the self. In a sense, it is a symbolic tool in which internal insecurities related to crises in housing, jobs, and even climate can be externalized in ways that allow people to maintain a sense of control in an uncontrollable world (Lindgren, 2009).

Beyond identifying a criminal other, looking at the migration theme reveals a common identity of the dangerous and unwanted other. Specifically, the group identifies Venezuelan migrants using terms like “rat,” “pest,” “plague,” and “parasite.” Previous studies have showcased the existing social anxiety among Colombians in the increasing migration of Venezuelans, noting them as increasingly unwelcome to the country (Bellino and Ortiz-Guerrero, 2023). Once again, this aligns with the role that race and nationality often play in discourses of good and evil concerning criminality or other unwanted activity (de Noronha, 2015). These discourses are not only present on social media platforms – they have a long history of being present in how media covers criminality and race (Aguilar, 2024; Webster, 1997). Positioning of us versus them is a common component of “prejudicial talk” and has been noted in past studies of digital platforms going back to online discussion boards (Rowe and Goodman, 2014). In these cases, the powerful or dominant group has the authority to label the *other* or non-dominant group, and often, such labeling can give an observer clues to the group’s composition, even in an anonymous forum like Telegram.

Finally, while it is not possible to capture the demographic composition of the Telegram group, a reading of the identities expressed in the messages of the group shows the presence of people across the political and gender spectrum, albeit with limited presence of migrants or those typically positioned as criminals. In this sense, O’Meara et al. (2024) show how people in digital communities engage in antisocial behavior by strategically operationalizing their identity “to normalize and legitimize their own practices as well as to undermine their targets and critics” (p. 7077). In this line of research, our findings foreground how intersectional identities shape how individuals relate to others in contexts of power (Rice et al., 2019; Shields, 2008) and how toxic speech is used in such positionality.

### Political (dis)affiliation and gender and sexuality

Concerning political (dis)affiliation, toxic speech was used to separate the group within ideological borders. However, in contrast to the discourses around (in)security, political (dis)affiliation remained much more contested. Indeed, we could not identify a single political affiliation that accurately represented the group. Participants in the group seemed to take toxic political speech in stride, or at least the presence of toxic speech did not seem to discourage users from pushing back on political viewpoints they found objectionable. This kind of discursive exchange is not without precedent. Researchers have found that, particularly in oppressive political regimes, communicative aggression is important for pushing back against oppression and increasing solidarity (Bodrunova et al., 2021). Although Colombia is a democratic government with relatively free and fair elections, The Freedom House (n.d.) indicates that outlaw groups sometimes impede the ability of citizens to participate free from harassment or intimidation, meaning there may be a good reason citizens would engage in toxic acts of social positioning, specifically concerning politics – a situation has been noted as possibly leading to a new wave of armed conflict in the country (Gutiérrez-Sanín, 2020). Other research suggests that aggressive political speech may be related to a struggle for power (Kaufova and Kaufova, 2021). Interestingly, there is no similar power struggle related to the role of migrants or the good-evil binary, which also gives clues as to who makes up the majority of active participants in the Telegram group.

The gender and sexuality narratives are more contested than the migration or (in)security narratives. However, the toxic speech tended to fall along predictable lines. In these cases, we see gender-based violence of men against women being called out in the group, where toxic speech is used to label violence against women as unacceptable. However, toxic speech concerning LGBTQ+-related violence serves to identify the LGBTQ+ community as unacceptable to others, positioning them as other and denying them group membership and protection. Like racist discourses, the othering of members of the LGBTQ+ community is not new and often stems from dominant media or political messaging (Montiel-McCann, 2023). Here, it hearkens back to a right-wing, pro-natalist, pro-heterosexual norm, so it can easily co-exist alongside the denouncement of violence against women. Far-right discourses around the world tend to co-opt the language of women’s rights while also being racist, anti-LGBTQ+, and anti-feminist in nature (Santos and Roque, 2021). In this sense, it might not be hypocritical for communities on Telegram to protect some community members while attacking others. Violence against women is a tool, just as (in)security is a tool from which to gain a sense of control and a sense of justification for the other bad behaviors of the group.

In light of these findings, how can we make sense of the societal implications of toxic speech? The uses of toxic speech as a positioning act differed across all four narratives in the sample. Even as research has long shown that antisocial behavior can only be fully understood within the confines of the specific contexts in which it is enacted and experienced, it has mostly emphasized how it is bounded by geography (Hawdon et al., 2017; Schoenebeck et al., 2023). In this case, our research points to the importance of placing antisocial behavior within the social, cultural, and communicative narrative contexts – that is, within the storylines that people have concerning specific issues or people. This view aligns with the work of Pohjonen and Udupa (2017) and Cover (2022), who emphasize the necessity of understanding antisocial behaviors within the ebbs and flows of everyday digital cultures. Thus, taking digital manifestations of violence out of the narratives they are aligning with or against results in incomplete readings of such antisocial processes. Toxic speech is not simply about putting someone else down; it is also about building up oneself and the group. Accordingly, in contexts where people feel a sense of insecurity – whether from criminality, rising inflation, or a global pandemic – we are more likely to find toxic forms of speech as people try to gain a sense of control (Chouliaraki, 2021).

Furthermore, the processes of othering and harming in this study showcase the need to clearly enunciate the logics of power embedded in the identities present and represented within specific storylines where toxic speech is used. Far-right speech is arising in contexts where a dominant group is feeling threatened by changes that impact their historical status. This is why we see migrants and LGBTQ+ individuals often targeted by toxic discourses from these communities. That said, like gossip itself, toxic speech on the Telegram group serves multiple purposes: it is an attempt at social control (Merry, 1984), it is an articulation of community and cultural norms (Gabriels and De Backer, 2016), and it is also a weapon aimed at marginalized communities. Overall, toxic speech in *Chismes Frescos Medellin* reveals efforts at reinforcing the power of a likely dominant group by dehumanizing others. It also happens to be a space where people find a community of sorts.

This study shows that a complete understanding of toxic speech must take into account both the social gratifications that interlocutors get from the positioning that the toxic speech affords and also the power imbalances, anxieties, and stressors that lead a group to want to position themselves in opposition to others to gain a sense of control. Indeed, when studying toxic speech, an ethical analysis of the structures of power must preclude all analysis of its social processes (Jane, 2015). In this sense, positioning theory offers a useful theoretical lens to surface these concerns, highlighting that it is not just the speech acts that matter but the work they are doing to define the boundaries of a community, who is worthy of being accepted by the community, and finally, which community deserves to be offered protection, care, and consideration. Toxic speech is both about the target of the speech and about the originator of the speech, and so we cannot start to understand how best to address and mitigate toxic speech until we first understand the different gratifications it gives those people who engage in it.

## Conclusion

In this article, we set out to examine the role that toxic speech plays in structuring processes of in- and out-group formation and maintenance. Our study demonstrates that toxic speech, like toxins (Tirrell, 2017), functions in complex and paradoxical ways. On one hand, the use of toxic speech to harm and position the *other* as outside one’s in-group contributes to the progressive deterioration of the social fabric that bonds diverse groups. This negative process was most clearly evidenced when Telegram group members engaged in xenophobic attacks against migrants, dehumanizing them and rejecting their right to inhabit Medellin. On the other hand, toxic speech functions as a mechanism to negotiate the positioning of normative boundaries, enabling users to claim belonging and express solidarity in prosocial ways. This enactment of toxic speech was most clearly evidenced when users used toxic speech to signal their capacity or intention to intervene in incidents of gender-based violence. Rather than emphasizing the destructive capacities of antisocial behavior, these instances illustrate how toxic speech can be a form of insurgence, solidarity, and resistance (Sundén and Paasonen, 2018; Tovares, 2018).

Unfortunately, we find that these practices of insurgency, solidarity, and resistance do not seem oriented toward structural changes that empower those most affected by toxic speech; rather, the Telegram group primarily uses toxic speech to reinforce existing power structures. These findings are consistent with Davis (2023), who argued that empathy and solidarity are often used as mechanisms for “othering,” with empathy being used “as a cure for structural issues without critically engaging its limits or allowing for other means of affective engagement” (p.2). Framed in this way, even statements communicating a capacity or willingness to intervene (potentially violently) as a bystander do not, in fact, aim to change the prevailing conditions (power structures) that enabled the harm to exist in the first place – instead, they normalize them.

Through this analysis, our study explored how toxic speech is used within a Colombian Telegram group to position oneself and others within narratives of political (dis)affiliation, (in)security, migration, and gender and sexuality. In line with previous work that has engaged with positioning theory to grasp the discursive construction of the self and others in contexts of violence on digital platforms (Badarneh and Migdadi, 2018; Labben, 2022; Sadaf and Siitonen, 2022), we have shown how toxic speech does more than just harm others. First, it is a way for social media users to mark who belongs (and who does not) to specific communities. Second, it helps to negotiate the normative boundaries of the group, drawing borders on what is allowed (or not) in the chat. Third, it represents a negotiation of which social groups are empowered and which ones are marginalized. Through this look at the contexts of toxic speech, our findings provide insights to better comprehend the social processes underlying antisocial behaviors on digital platforms.

Given the limited scope and findings of this study, there are opportunities for future research. First, as this study is based on a single case study, future studies could explore toxic speech as positioning acts across different technological and geographical contexts. Second, given the critical role of identity in the process of positioning and the technical limitations in obtaining demographic information on participants, future studies could take a closer look at how intersecting identities play a role in shaping the use of toxic speech to position oneself and others. Third, future scholars could further examine the role of particular affordances that facilitate and shape positioning acts – by exploring, for example, whether people use Facebook’s comments or Instagram’s reactions in similar ways as displayed in this study. Finally, our findings call for interventions that take into consideration the double-bind dilemma of toxic speech – that is, interventions that support its pro-social uses and foster belonging while reducing its antisocial mechanisms of othering. Here, we might imagine a number of possible interventions which could be tested, such as encouraging bystander intervention in support of a marginalized group (e.g. Ringrose et al., 2025) or governance mechanisms (content moderation guidelines or algorithmic sorting) that foster more healthy ways for the community to grow and connect (e.g. Viejo Otero, 2024). Future work should explore these and other interventions.

Our study has important implications for those interested in exploring and addressing antisocial behaviors on digital platforms. For instance, this study has implications for those exploring the role of content moderators. Indeed, in light of our findings, the content moderators’ role is not to impede toxic speech under a blanket. Instead, it is to determine the ethical boundaries of the narratives that prevail on the groups (e.g. we can discuss whether or not we agree on migration, but we cannot wish them dead). In addition, our study has important implications for those seeking to grasp public discourse in online spaces in a contemporary polarized landscape. For instance, our findings showcase possible reasons why people are less likely to want to moderate toxic speech when it occurs in their in-groups. Overall, our findings invite researchers of antisocial behavior to pay closer attention to the contexts of toxic speech, noting the processes of belonging and othering that underlie online abuse and hate.
